# Unpacking WHO and CDC Bottle Bioassay Methods: A Comprehensive Literature Review and Protocol Analysis Revealing Key Outcome Predictors

**DOI:** 10.12688/gatesopenres.15433.2

**Published:** 2024-12-18

**Authors:** Giorgio Praulins, Annabel Murphy-Fegan, Jack Gillespie, Frank Mechan, Katherine Gleave, Rosemary Lees

**Affiliations:** 1 Innovation to Impact (I2I), Department of Vector Biology, Liverpool School of Tropical Medicine, Liverpool, L3 5QA, UK

**Keywords:** Insecticide Resistance, CDC Bottle Bioassay, WHO Bottle Bioassay, Vector Control, Methodological Variability, Resistance Monitoring, Method Validation

## Abstract

**Background:**

Resistance monitoring is a key element in controlling vector-borne diseases. The World Health Organization (WHO) and Centres for Disease Control and Prevention (CDC) have each developed bottle bioassay methods for determining insecticide susceptibility in mosquito vectors which are used globally.

**Methods:**

This study aimed to identify variations in bottle bioassay methodologies and assess the potential impact on the data that is generated. Our approach involved a systematic examination of existing literature and protocols from WHO and CDC, with a focus on the specifics of reported methodologies, variation between versions, and reported outcomes. Building on this, we experimentally evaluated the impact of several variables on bioassay results.

**Results:**

Our literature review exposed a significant inconsistency in the how bioassay methods are reported, hindering reliable interpretation of data and the ability to compare results between studies. The experimental research provided further insight by specifically identifying two key factors that influence the outcomes of bioassays: mosquito dry weight and relative humidity (RH). This finding not only advances our comprehension of these assays but also underscores the importance of establishing precisely defined methodologies for resistance monitoring. The study also demonstrates the importance of controlling bioassay variables, noting the significant influence of wing length, as an indicator of mosquito size, on mortality rates in standardized bioassays.

**Conclusions:**

Generating data with improved protocol consistency and precision will not only deepen our understanding of resistance patterns but also better inform vector control measures. We call for continued research and collaboration to refine and build consensus on bioassay techniques, to help bolster the global effort against vector-borne diseases like malaria.

## Introduction

Insecticide-based vector control is one of the main tools in the fight against malaria ^1^ and so insecticide resistance monitoring is essential to inform operational decisions in vector-control. To be able to select appropriate vector-control interventions the insecticide resistance status of the local mosquito population needs to be well understood. Although the range of insecticide classes available for mosquito control is limited, a crucial element in the management of resistance is the ability to be able to switch between insecticide classes where resistance is seen to have evolved to a specific insecticide is observed. This approach helps address the issue of cross-resistance, ensuring that control measures remain effective against mosquito populations that have developed resistance to certain insecticides and particular modes of action (MoA) ^2^. Where a novel insecticide is introduced, it is important to be able to monitor for the emergence of resistance in the target mosquito population.

The CDC bottle bioassay, recommended by the Centres for Disease Control (CDC), was developed by Brogdon and McAllister in 1998 ^3^. It is a resistance monitoring assay which looks at time-to-kill of a population exposed to a specified concentration of an insecticide, with knockdown measured 10–90 minutes after exposure, dependent upon the tested insecticide ^4, 5^. It was developed as an additional means of monitoring resistance to the original World Health Organisation (WHO) tube bioassay, which measures knockdown within 1 hour of exposure and mortality24-hours post exposure ^6^.

The WHO method offers distinct advantages over the CDC method by standardizing test materials using centrally prepared, quality-assured impregnated papers, which ensures uniformity and reliability of results across various locations. This eliminates the inconsistencies associated with local preparation of bottle coatings. Moreover, the method minimizes hazardous exposure to insecticides for technicians by avoiding manual bottle coating processes. Additionally, the WHO method's logistical efficiency is enhanced through the use of easily transportable impregnated papers and plastic tubes, making it less cumbersome and more practical for field deployment compared to the more bulky and hazardous materials required by the CDC method ^3, 7^. Data generated using the assay is widely used for the routine monitoring of resistance to commonly used insecticides in field populations and for the characterisation of laboratory populations of mosquitoes ^5^.

The original CDC bottle bioassay test procedures ^3^ have been updated several times to reflect an evolving understanding of mosquito resistance phenotypes. Standardized guidelines published by the CDC offer detail on some parameters for conducting these assays, including recommendations on diagnostic dose (s), exposure time, and mosquito age ^8^, but there are some experimental parameters in the method which are open to interpretation as to how exactly to perform the bioassay such as number of mosquitoes per bottle, the environmental conditions and orientation of the bottles for testing. This introduces the potential for variability in how the bioassay is conducted between testing sites, and this potential for inconsistency is borne out by differences in methods used between studies as reported in publications and elsewhere.

If these methodological differences affect the results obtained from the bottle assay, data may not be comparable between sites, time points or insecticides, which could impact the precision and repeatability of the evidence on which operational decisions are made. The age of mosquitoes when exposed to insecticides can have an impact on measured susceptibility, with a decline in resistance observed in older mosquitoes in comparison to younger ones ^9– 11^. The number of mosquitoes per bottle and agitation of the bottle during testing, for example during scoring knockdown/mortality, could disrupt resting behaviour, impacting contact time with the active ingredients and subsequent insecticide exposure, as observed in the WHO tube bioassay ^12^. Bottles used for bioassays are typically coated manually either where the tests are conducted or at a collaborating institute before being shipped. This manual coating process might introduce extra variability compared to the World Health Organization (WHO) tube tests. In the WHO tube tests, insecticide-impregnated papers are produced and distributed from a central location, where they are also hand-treated ^13^. Interpretation of the bottle coating technique and how evenly coated insecticide is could vary between users, and prolonged or frequent contact with a surface that has not been evenly coated could impact mortality results. Environmental factors can also affect the resistant phenotype. Low humidity has been shown to potentially increase insecticide-induced mortality in mosquitoes ^14^. On the other hand, higher temperatures have been correlated with varied effects: an increase in susceptibility to insecticides in some instances, while in others, they have been associated with improved mosquito longevity ^15– 17^. Therefore, it is important to conduct susceptibility bioassays consistently, controlling conditions as closely as possible and reporting in detail the methodological parameters and environmental conditions.

The development of the WHO bottle bioassay method ^7, 18– 20^ was influenced by the challenges encountered in establishing diagnostic concentrations (DCs) for new classes of insecticides, specifically the neonicotinoid clothianidin and the pyrrole chlorfenapyr. Susceptibility to these insecticides has been measured using various testing methods by separate groups. For instance, clothianidin has been tested by impregnating filter papers with SumiShield™ 50WG ^21^, and both insecticides have been tested in CDC bottle bioassays ^22^. Comparative studies have used both CDC bottles and SumiShield™ 50WG impregnated filter papers side by side ^23^. This variation in methodology, coupled with the use of different insecticidal products or technical grade insecticides, make it difficult to compare results from these early studies. Recognizing this challenge, the WHO included these and other chemistries of interest in their discriminating dose study, which has now led to their recommendation of the WHO bottle bioassay method for both insecticides ^7, 19^. The CDC bottle bioassay similarly sought to overcome the issues of stability on papers by instead coating a glass surface with insecticide. The method measures the time to knockdown (or incapacitation) of mosquitoes exposed to a specified dose of insecticide, in contrast to the endpoint measured by the WHO bottle bioassay, mosquito mortality 24 hours after a 1-hour exposure. Measuring 24hr mortality allows closer comparison with susceptibility in the WHO tube assay and provides more consistency between methods across the WHO guidelines, streamlining testing procedures for country teams ^7^. As novel insecticidal compounds continue to emerge, the WHO bottle bioassay will play a crucial role in monitoring susceptibility to ensure their effective deployment in public health initiatives ^20, 22^.

To try to understand how consistently each bottle bioassay methodology is being applied, we set out to review the current literature for instances where the guidelines are being referenced, to see what data and information is being reported, and where there are gaps or inconsistencies in method reporting. We then aimed to experimentally explore parameters of the bioassay we found not to be consistently reported between studies, to determine their impact on bioassay results, and where appropriate suggest tighter ranges of values for these parameters to minimize variability in data. We aimed to do this by looking for parameters of the guidelines which are open to interpretation and seeing if they can influence the results of the bioassay. In doing this we hope to suggest additional guidance on optimal performance of this bioassay and key information required for reporting of insecticide resistance data, thus proposing a model for more robust data generation and reporting using this methodology.

## Methods

### Guidelines review: assessing updates and changes in methodology

The original method published by Brogdon and McAllister (1998) and subsequent CDC documentation containing methods for the CDC bottle bioassay or the rationale behind the bioassay parameters were reviewed to compare specific methodological detail. The documents identified for review were:

- “Simplification of Adult Mosquito Bioassays Through Use of Time-Mortality Determinations in Glass Bottles” Brogdon and McAllister (1998) ^3^
- “Guidelines for Evaluating Insecticide Resistance in Vectors Using the CDC Bottle Bioassay” CDC (2010) ^8^
- “CONUS Manual for Evaluating Insecticide Resistance in Mosquitoes Using the CDC Bottle Bioassay Kit” CDC (2016) ^4^
- “Manual for Evaluating Insecticide Resistance Using the CDC Bottle Bioassay” CDC (2023) ^5^ Additionally, the guidelines which outline the new WHO bottle bioassay method were considered:
- “Standard operating procedure for testing insecticide susceptibility of adult mosquitoes in WHO bottle bioassays” WHO (2022) ^18^

The WHO bottle assay method is an adaptation to the CDC bottle bioassay where time to kill scoring is no longer done and instead the method resembles that of the WHO tube bioassay where mosquitoes are exposed to a set diagnostic dose with a set exposure time and then scored at either 24 hour or a later time point for mortality (or oviposition inhibition in the case of pyriproxyfen). Diagnostic doses pre-established by CDC for older insecticides as well as new doses for newer insecticides identified from the WHO multicentre study “Determining discriminating concentrations of insecticides for monitoring resistance in mosquitoes: report of a multi-centre laboratory study and WHO expert consultations” are included ^19^.

For each of these documents, methodological details were extracted and then compared, other than the CONUS Manual for Evaluating Insecticide Resistance in Mosquitoes Using the CDC Bottle Bioassay Kit, 2019 method ^4^ which was published by the CDC as an update to the guidelines produced in response to the Zika epidemic and so was largely unchanged from the 2010 version, the major difference being updated doses and times for a range of mosquito vectors that are found in the continental U.S. ^4^. Since this guideline focuses on testing *Aedes* mosquitoes and was not comparable to the others it was excluded from the review.

An outline of the differences between these methods documents is detailed in Table 1, which lists all the parameters that were reviewed and any differences between the guidelines. Several areas were identified that are open to interpretation, dependent on which published method is followed by the operator, which could affect the knock down or mortality score and so reduce comparability between studies.

**Table 1.** Key differences between published methodologies for bottle bioassay testing.

Category	Methodological Parameters	Brogdon and McAllister 1998 ^3^	CDC 2010 ^25^	CDC 2023 ^5^	WHO 2022 ^18^
Characterising/preparing mosquitoes	Species identification	Not detailed	Carry out species identification before or after testing	Species identification before testing and conduct bioassay on distinct species separately	Not detailed
Selecting/preparing test item	Reagents to be tested	Not detailed	Technical grade and formulations	Technical grade only	Technical grade
	Generation of field mosquitoes	Not detailed	Not detailed	F1/F2 generation	Not detailed
	Mosquito age	3–4 days	Not detailed	2–5 days	3–5 days
Data analysis/interpretation	Abbott's correction	Not detailed	Control mortality 3–10%	Control mortality 3–20%	Control mortality ≥5% and ≤20%
	Number of mosquitoes per bottle/bioassay	25 (minimum 5)	100 across 4 replicate bottles. 10–25 control bottle	100 across 4 replicate bottles. 10–25 control bottle	25
	Bottle coating method	Manual	Manual	Manual and use of bottle rollers	Manual
	Cleaning protocol	Not detailed	Rinse with warm soapy water and tap water	24-hour soak in hot water with dish soap. Advice to test cleanliness using 5–10 susceptible female mosquitoes	Soak in Decon overnight and in water for 24 hours
	Insecticide type	Not detailed	Technical grade and formulation	Technical grade only	Technical grade
During testing	Orientation during testing	Not detailed	Not detailed	Horizontal or vertical	Vertical
	Shelf-life of stock solutions	Not detailed	2–3 years	2–3 years pyrethroids and carbamates, 24-hour shelf life for organophosphates	Not detailed
	Use of synergist	Use of PBO and DEF mentioned	Details preparation, testing instructions and concentrations	Details preparation, testing instructions and concentrations	Details preparation, testing instructions and concentrations
	Synergist testing: PBO concentration	Not detailed	400µg/bottle	100µg/bottle	Not detailed
	Mosquitoes per bottle for PBO testing	Not detailed	125	25	Not detailed
	Recommend stirring/ agitation during test setting	Rotate bottle during mortality scoring	Rotate bottle during mortality scoring	Rotate bottle during mortality scoring	Not detailed
	Recommendations for behavioural assessment	Not detailed	Not detailed	Not detailed	Not detailed
	Species specific recommendations	Not detailed	Diagnostic dose and times given for Anopheles and Aedes	Diagnostic dose and times given for Anopheles and Aedes	Diagnostic doses given for Anopheles and Aedes
	Insecticide concentrations	Not detailed	Diagnostic doses given	Diagnostic doses given	Discriminating concentration given
	Insecticide exposure time	Not detailed	Diagnostic times given (30–60 minutes)	Diagnostic times given (30–60 minutes)	1 hour
	Number of mosquitoes required per treatment evaluation	Not detailed	100	100	150

### Literature review: investigating methodological inconsistencies in performing or reporting on bottle bioassays

To identify publications that reported using the bottle bioassay to compare reported methodological detail, PubMed and BioMed Central databases were searched on January 2021 using the search terms “mosquito”, “CDC” and “bottle”. Another search was done in August 2023 to identify any additional papers that had been published since the initial search. Using these search terms, 177 results were returned from PubMed and 74 from BioMed Central. Duplicates were excluded and 103 papers were screened and excluded if they were not relevant to the CDC bottle bioassay or did not test on mosquitoes. Further papers were excluded based on the following criteria:

- Studies where the MCD bottle bioassay was used. This low-cost method tests insecticides using a modified 1-L plastic bottle with netting, into which mosquitoes are introduced. It evaluates transient contact effects and behavioural responses to various insecticide formulations, making it not comparable to the CDC or WHO bottle bioassay ^24^.
- Mosquito age not reported.
- Knockdown/mortality data not included.
- Number of mosquitoes used not reported.
- An insecticide was not tested.
- Mortality results were based on assays where the mosquitoes had been previously microinjected.
- Incorrect referencing of bioassay test procedures (e.g., an academic publication or other documentation which did not provide a fully outlined protocol for the bioassay).

On these criteria a further 72 papers were excluded. Additionally, two papers were identified as duplicates present in both databases, and their duplicate entries were removed. This resulted in a total of 74 papers, from which the following information was extracted:

- Mosquito age during testing.
- The methods document referenced.
- If there were any mention of behavioural assessment.
- Any details on bottle coating and on bottle drying.
- Country of testing.
- Insecticide tested and concentration.
- Exposure time to insecticide.
- Holding time post insecticide exposure.
- Knockdown recording interval used to calculate time to kill.
- Orientation of bottle during testing - vertical or horizontal.
- Whether bottle was kept still during exposure or agitated.
- Generation of field mosquitoes used – number of generations between collection of wild mosquitoes and bioassays.
- Use of synergists such as MERO (rapeseed oil methyl ester, prevents crystallisation of insecticides on a glass surface).
- Sample size - number of mosquitoes per bottle, per treatment in 1 bioassay, and per control.

**Figure 1.**
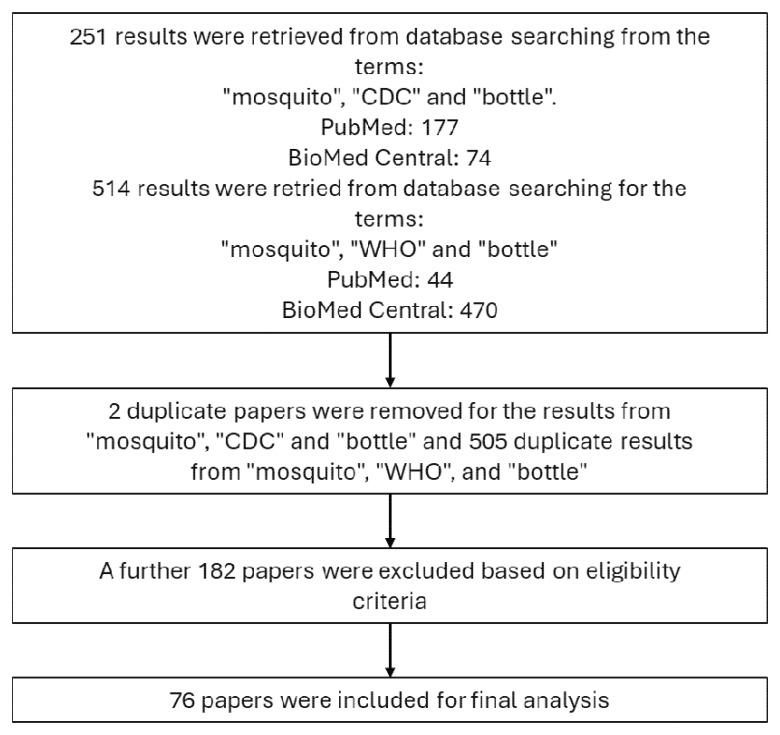
Flow diagram describing the methodology of the literature review.

A secondary search of “mosquito,” “WHO” and “bottle” was also carried out to identify any papers published which reference the 2022 WHO guidelines “Standard operating procedure for testing insecticide susceptibility of adult mosquitoes in WHO bottle bioassays.” 44 results were returned on PubMed, and 470 from BioMed Central. 505 duplicate papers were removed which had been included in the previous searches for references to the CDC bottle bioassay and 7 papers were excluded, which left 2 additional publications to be included in the analysis, totalling 76 included papers, as detailed in Figure 1. The same information was extracted from these publications as listed above.

### Experimental investigation: understanding the level and sources of variability in results from the WHO bottle bioassay

Our project's primary goal was to quantify and identify the sources of variation in the WHO Bottle bioassay, focusing not just on standardization and technician training, but also on how these factors interact under controlled climatic conditions in a laboratory setting. This approach allowed us to investigate the assay's variability even when external variables were minimized. We focused on the WHO bottle bioassay ^18^, which consists of a single exposure with ≥24-hour mortality scoring in line with WHO tube methodology, since it is a novel method which is likely to be widely adopted for susceptibility monitoring in Anopheles, Aedes and Culex mosquitoes as well as sandflies ^18, 26^. We therefore measured variation in 24-hour mortality as per the WHO bottle bioassay method, rather than on the many scores of knock down which are used to determine time to kill in the CDC bottle bioassay. We anticipated that there would be less variability in the 24-hour mortality endpoints than in the knockdown measurements made every few minutes during a time to kill bioassay. This enabled us to design experiments with the necessary precision to effectively assess the impact of various experimental parameters on the entomological endpoint. Mortality was measured in a susceptible strain of mosquitoes, Kisumu 24 hours after exposure to a standard pyrethroid. We either kept parameters controlled or monitored parameters to assess them as sources of variability (See Table 2).

**Table 2.** Experimental parameters controlled or monitored as part of the experimental work of this study.

Category	Parameter	Details
**Controlled**	Bottle use (number of individual uses)	Used once
	Bottle use (time)	Used within 24 hours
	Exposure length	1 hour
	Feeding status	Sugar fed
	Holding time	24 hours
	Humidity	80 ± 5%
	Insecticide	Permethrin
	Insecticide Concentration	0.48µg per bottle
	Light cycle	L12:D12 hour light: dark cycle and a 1-h dawn and dusk
	Mating status	Mated
	Mosquito age	3–5 days
	Number per test unit	~25 (20–35)
	Operator	Experimental work carried out by both or one of two operators
	Scoring timepoints	1 hour and 24 hours
	Sex	Female
	Strain/Species	*Anopheles gambiae* (Kisumu [Susceptible])
	Temperature	26 ± 2 °C
**Monitored**	Size (wet weight/dry weight)	Measured in grams
	Date of test	Recorded on the day
	Knockdown/Mortality	Recorded on the day
	Mosquito Strain Generation	Recorded on the day
	Size (wing length)	Measured in mm

The outputs from these assessments enabled us to determine the precision, repeatability (intra-assay precision), intermediate precision (inter-assay precision), of the WHO bottle bioassay under controlled conditions.

***Mosquito rearing.*** The Kisumu strain of *Anopheles gambiae*, a reference insecticide-susceptible strain, was used for all experiments. Mosquito colonies were maintained as described by Williams *et al*., in the Liverpool Insect Testing Establishment (LITE) facility at the Liverpool School of Tropical Medicine (LSTM) ^27^. Insectary conditions were maintained at 26 ± 2 °C and 80 ± 5% relative humidity (RH), with a L12:D12 hour light: dark cycle and a 1-h dawn and dusk. The larvae were reared in purified water and fed ground TetraMin ^27^. Adults were provided continuous access to a 10% sucrose solution and adult females were given the opportunity to feed on human blood, composed of blood plasma and red blood cells supplied by the human blood bank and mixed upon arrival at LSTM. For the feeding process, a Hemotek membrane feeding system, provided by Hemotek Ltd based in Blackburn, UK, was used.

***WHO bottle bioassays.*** In order to measure the consistency and reliability of measurements of mortality in the WHO bottle bioassay, repeated tests were performed under the same conditions and by the same two operators, and a median lethal concentration (LC _50_) for permethrin, a concentration expected to kill an average of 50% of exposed Kisumu mosquitoes. Permethrin was chosen as a representative pyrethroid insecticide. The aim was to measure the minimum or inherent variability in the bioassay, with as much standardisation as possible and so for this reason LC _50_ was chosen as it would allow for the greatest amount of variation to be observed above and below this value. Two phases of bioassay testing were done, with the methodology for phase 2 adapted based on preliminary findings from phase 1:

- Phase 1: In 2022, two operators each tested seven units (bottles) per day across nine days, resulting in 14 test units per day. This approach aimed to identify any significant differences in results due to operator variability.
- Phase 2: Observations from 2022 showed no significant operator-dependent differences, leading to a methodological adjustment in 2023 to a single operator conducting all bioassays each day. Across 12 days, 12 bioassays were performed, each comprising seven test units. Due to a dilution error, one set of test units was excluded, resulting in the use of five bioassays from one operator and six from another for analysis.

A 'biological replicate' refers to a single test unit (bottle), while a 'technical replicate' encompasses a set of bioassays (7 bottles) conducted on the same day.

Based on historical experimental data from another research group who had used the Kisumu colony for a previous unrelated study (unpublished data), this study was started with an LC _50_ of 12.5µg of permethrin per bottle. However, 100% mortality was recorded in these initial bioassays, and a preliminary dose response experiment was required to establish a ‘true’ LC _50_. Each operator conducted a separate dose response experiment with permethrin concentrations of 0.1µg, 0.5µg, 1µg, 2.5µg, 5µg, 7.5µg, 10µg, and 12.5µg per bottle. Combining results from these two tests (one per operator), a probit analysis in PoloPlus (Version 2.1, LeOra Software) established the LC _50_ as 0.48µg per bottle ^28^.

For each bioassay, bottles were coated with either permethrin dissolved in acetone at the determined LC _50_ (0.48µg per bottle) or with acetone alone for control. The bottles were glass 250mL volume Wheaton media bottles fitted with PTFE-lined lids (Sigma-Aldrich, Merck KGaA, Darmstadt, Germany), were manually rolled and inverted to ensure even coating of all internal surfaces, including the lid ^8, 18^. After removing the lids, bottles were left on a roller overnight to dry at ambient temperature (20 and 25 °C).

In each bioassay, approximately 25 (within the range 20–35) non-blood fed female mosquitoes, aged between 3 and 5 days, were held in 10 ml pipette tips plugged with cotton wool to acclimatize to the test laboratory, then introduced into the bottles for a one-hour exposure to the insecticide.

Approximately 25 additional mosquitoes were collected to measure their weights and wing lengths. To determine their weights, the mosquitoes were first incapacitated by placing them in a freezer for 30 minutes. Afterward, they were weighed on a Sartorius Balance (Model TE214S), and this total weight was divided by the number of mosquitoes to calculate an average "wet weight." This was used as a representative measure for the mosquitoes used on that day of testing. To obtain their "dry weight," the mosquitoes were desiccated by storing them in a 25mL falcon tube filled with silica gel, which served to remove moisture, and cotton wool, which prevented direct contact between the mosquitoes and the silica gel. This setup was left undisturbed for one week to ensure the mosquitoes were completely dried out before they were weighed again. The total weight was then divided by the number of mosquitoes to determine the average "dry weight." This was used as a representative measure for the mosquitoes used on that day of testing.

For the wing length measurements, wings were dissected from a subset of 10 mosquitoes. These wings were examined under a GXMMZs0745 microscope (GT Vision, UK) and images were captured using a GXCAM Eclipse Camera (GT Vision, UK) and the GXCAM software (Version 6.7, GT Vision, UK). The images obtained were analysed with ImageJ (Version 1.54d, NIH, USA) to measure the mosquito wing lengths from apical node to vein number 10 ^29^. This was used as a representative measure for the mosquitoes used on that day of testing.

Post-exposure, mosquitoes were transferred using a vacuum pump into paper cups covered with netting, provided with a 10% sugar solution and held for 24 hours before mortality was scored. Mortality was additionally scored at 1-hour post-exposure but was not included in the analysis due to prominent levels of variability above that of 24-hour mortality.

Testing conditions were held as constant as possible to measure the ‘inherent’ variability of the bioassay with as few external sources of variability as possible. Bioassays were performed under controlled conditions set at applicable control limits of 26 ± 2 °C temperature and 80 ± 5% RH, with precise parameters recorded at the start and end of each testing session. This was recorded through the Building Management System (BMS) which continually monitors and controls the environment of the test facility. If the testing areas fall outside of their applicable control limits, an alarm is generated. Data on temperature and humidity is recorded at intervals by the system and can be accessed and retrieved as required. During both testing and holding periods throughout the full run of the experiment the temperature and humidity did not fall outside of the applicable control limits. All bioassays were conducted between 2pm and 3pm to minimize variability as a result of circadian differences in mosquitoes. Fresh insecticide stock was prepared for each test to ensure consistency, and all bottles were used for testing only once and within 24 hours of coating. Operators scored mortality independently to avoid bias. Each operator conducted seven technical replicates with pyrethroid-treated units and two with acetone-only controls. Tests were repeated over multiple weeks using different mosquito cohorts for further biological replication.

***Data analysis.*** Associations between parameters of interest and outcome (24hr mortality) were assessed using Generalised Linear Mixed Models (GLMMs) using the ‘lme4’ package in R. The response variable was the outcome of interest (24hr mortality), included as a binomial outcome (Dead or Alive). Replicates where control mortality exceeded 20% were excluded from the analysis and control mortality in the analysed data ranged between 0 and 20%. To quantify unexplained variation between assays, a random effect term was included for both technical and biological replicate. Parameters being assessed were included as either a categorical variable (‘operator’) or a continuous variable (‘wing size,’ ’dry weight,’ ’number of mosquitoes per bottle,’ ’temperature,’ ’humidity’). The statistical significance of each parameter being assessed was evaluated using log likelihood ratio tests, where the explanatory power of a model with the parameter included was compared to an equivalent one without. As the purpose of the study was to quantify and explain variability in observed outcomes, it was decided to assess the true outcomes rather than an adjusted metric and so an Abbot’s correction was not used for this study.

## Results

### Guidelines Review: assessing updates and changes in methodology

The CDC bottle bioassay was developed in response to alterations to the WHO tube bioassay methodology. The original WHO tube methodology ^30^ exposed field collected mosquitoes to a range of insecticide concentrations and then probit analysis was carried out on the data generated. In the 1980’s this test was modified to use only papers of a single known concentration due to the difficulty of collecting sufficient field mosquitoes suitable for testing ^31^. At the time this new WHO methodology had several drawbacks: the test kit was seen to be expensive; test papers were not provided by WHO for certain insecticides; there was no provision for assessment of metabolic resistance; and the recommended diagnostic doses were not appropriate for all species. As a result, CDC looked to develop a bioassay methodology to address these issues ^31^.

Brogdon had previously published work using a microplate-based biochemical assay ^32^, and this method was adapted to use insecticide coated 250 mL glass Wheaton bottles and published in the Journal of the American Mosquito Control Association in 1998 ^32^. The original bioassay method outlined by Brogdon & McAllister ^3^ exposed 25 3–4-day old non-blood fed female mosquitoes to 250 mL glass Wheaton bottles coated with a known concentration of insecticide and mortality was scored at regular intervals until mortality reached 100%. The findings of this paper were then developed into a set of guidelines for performing the CDC bottle bioassay. However, there were some changes made to the protocol for the purposes of these guidelines. The published guidelines suggest that 10–25 female mosquitoes of known age and physiological status are exposed to four replicate bottles, with a minimum of one hundred mosquitoes per test concentration and an additional bottle with 10–25 mosquitoes as a control. This is slightly open to interpretation however as the guidelines specifically quote that “results of multiple bioassays over a few days may be pooled to achieve the recommended sample size, 100 mosquitoes. In either case, each bioassay must include a control bottle with 10–25 mosquitoes”. This lack of clarity could result in variability in the quantity of mosquitoes exposed per test bottle within and between studies ^8^. The bioassay in the guidelines is otherwise conducted the same as outlined in the publication, with knockdown being scored at regular intervals until knockdown/mortality reaches 100%. Beyond these details the CDC methodology does not give many more specifics for how to perform the bioassay, for example there is no mention of the environmental conditions the test should be performed at or the orientation of the bottles during exposure ^8^.

Two additional documents were published to supplement the CDC (2010) guidelines. The first was “Insert 1 – Revised Box 5: Interpretation of data for resistance management purposes” and was published in 2012. This document gave specific information for the interpretation of mortality rates in insects to infer insecticide resistance, laying out benchmarks for when further testing or action is needed, and emphasizing the importance of proactive measures to manage confirmed resistance and preserve the effectiveness of insecticides for malaria vector control ^33^. In 2013, the document titled "Insert 2 - Enhanced Surveillance Protocol for the CDC Intensity Bottle Bioassay" was published as a direct response to the shortcomings of traditional methods such as the WHO tube assay and the CDC bottle assay which primarily focus on determining the frequency of insecticide resistance using a standard discriminating dose. However, through extensive field experiences, it became evident that merely assessing resistance frequency was insufficient for making crucial decisions in insecticide procurement and deployment strategies. Recognizing this gap, the new protocol introduced the concept of assessing resistance intensity, crucial for a more comprehensive understanding of insecticide resistance strength. The WHO also started to recommend that higher doses be used to evaluate resistance, 5x and 10x the diagnostic concentration ^6^. The insert outlined methods for the effective collection of adult mosquitoes using backpack aspirators and described three Rapid Diagnostic Tests (RDTs) — Resistance Frequency (F-RDT), Resistance Intensity (I-RDT), and Resistance Mechanism (M-RDT). These tests were designed to provide a holistic view of resistance, enabling more effective and cost-efficient strategies for resistance management. By incorporating tests that measure not only the frequency of resistance but also its mechanisms, both protocols offered a more detailed and actionable insight into the resistance profiles of mosquito populations. The emphasis on resistance intensity was a novel approach that reflected a shift in focus from merely identifying the presence of resistance to understanding its severity and implications for vector control. This shift was critical for decision-making processes, especially in areas where mosquitoes exhibited varying resistance levels to different insecticide doses. Overall, the insert represented a proactive and necessary response to the evolving challenges in mosquito resistance management, aligning with the growing need for more nuanced and detailed resistance assessment methods as suggested by global health authorities like the WHO ^34^.

In the updated 2023 version of the "Manual for Evaluating Insecticide Resistance Using the CDC Bottle Bioassay" ^5^, the overall protocol remained largely the same however it does introduce several significant changes compared to its 2010 predecessor. One of the primary alterations is in the terminology. The updated version emphasizes the bioassay as an early-warning system for insecticide resistance, requiring intervention if susceptibility changes. Changes in scope between the new manual and older guidelines are listed below and outlined in Table 1:

- In terms of scope, the new manual now covers information on how to evaluate both the frequency and mechanisms of insecticide resistance.
- The 2023 guidelines subtly shift from the 2010 recommendations on insecticide selection for vector control by omitting the previously emphasized need for detailed bioassay evaluations to detect insecticide resistance. This change does not suggest a discouragement of bioassay use but rather introduces a more flexible approach to decision-making. It reflects an evolution in guidance, allowing programs to tailor their strategies to specific needs without prescribing a uniform preliminary step of conducting bioassays.
- While the 2010 guidelines allowed species identification before or after testing, the current guidance recommends conducting bioassays on distinct species separately and so this species identification needs to be done ahead of bioassays.
- The updated version reverses the earlier advice against using lab-reared mosquitoes, now providing detailed guidelines for utilizing F _1_ or F _2_ generations that are 2–5 days old.
- The phrase "vector populations" has been replaced with "mosquito populations" to improve specificity.
- The range for using Abbott's correction has been updated. While the CDC's 2010 guidelines specified a control mortality range of 3–10%, the CDC's 2023 guidelines have expanded this range to 3–20%. This is also in line with the new WHO 2022 bottle bioassay protocol which recommends applying the formula for control mortality at ≥5% and ≤20%. All guidelines recommend experiments are repeated if control mortality falls above the stated range for correction.
- Previously, the comparison to susceptible or baseline mosquito populations was recommended, which is now omitted.
- The assessment of resistance intensity has been moved to a new section and is now assessed by intensity bioassays, which consist of exposing mosquitoes to increasing multiples of a diagnostic insecticide dose to gauge resistance intensity more accurately.
- Minor changes have been made to the synergist bioassay section, reducing the concentration of PBO from 400μg/bottle to 100μg/bottle and updating the number of mosquitoes per bottle from 125 to 25.

The changes to the methodology between the old and new guidance are as follows:

- The bottle coating procedures have been expanded to include the use of bottle rollers, in addition to manual rolling.
- Cleaning protocols for the bottles have been updated. Instead of a simple rinse with warm soapy water and tap water, the new guidelines suggest a 24-hour soak in hot water with dish soap. Furthermore, the use of 5–10 susceptible female mosquitoes in a bioassay are now recommended for assessing the bottles' cleanliness.
- The 2023 version recommends testing only technical-grade insecticides, discontinuing the earlier option to test formulations.
- Guidance on the shelf-life of stock solutions has been refined. Whereas the older version stated that stock solutions could be kept for 2–3 years, the updated version specifies this only for pyrethroids and carbamates, limiting organophosphates to a 24-hour shelf life.
- The updated version provides more comprehensive information on safety precautions and waste disposal ^5^.

### Literature review: investigating methodological inconsistencies in performing or reporting on bottle bioassays

A review of papers referring to the use of the CDC or WHO bottle bioassays identified studies testing a range of Anopheline and Culicine mosquito species. In instances where more than one publication assessed the same mosquito strain and insecticide combination, an effort was made to compare the mortality data to determine the consistency of results between studies. In 30% of publications only field-collected mosquitoes were tested and so these papers were excluded from this analysis, because our experimental work was lab-focused, we were primarily interested in studies conducted on established lab strains. While we acknowledge the importance of field studies where bottle bioassays are typically deployed, we opted to exclude publications testing only field-collected mosquitoes due to the challenges associated with comparing different mosquito populations and the inadequately reported methodological details. Assessing inter-bottle variation in these studies would be a valuable line of future investigation. A further 41% of studies tested field populations alongside a susceptible laboratory reference strain, the majority being the *An. gambiae* strain Kisumu (41%). In those publications containing results from testing with the same strain and insecticide combination the agreement was good, with the reported mortality against DC’s being extremely high (>90%) in all cases.

**Figure 2.**
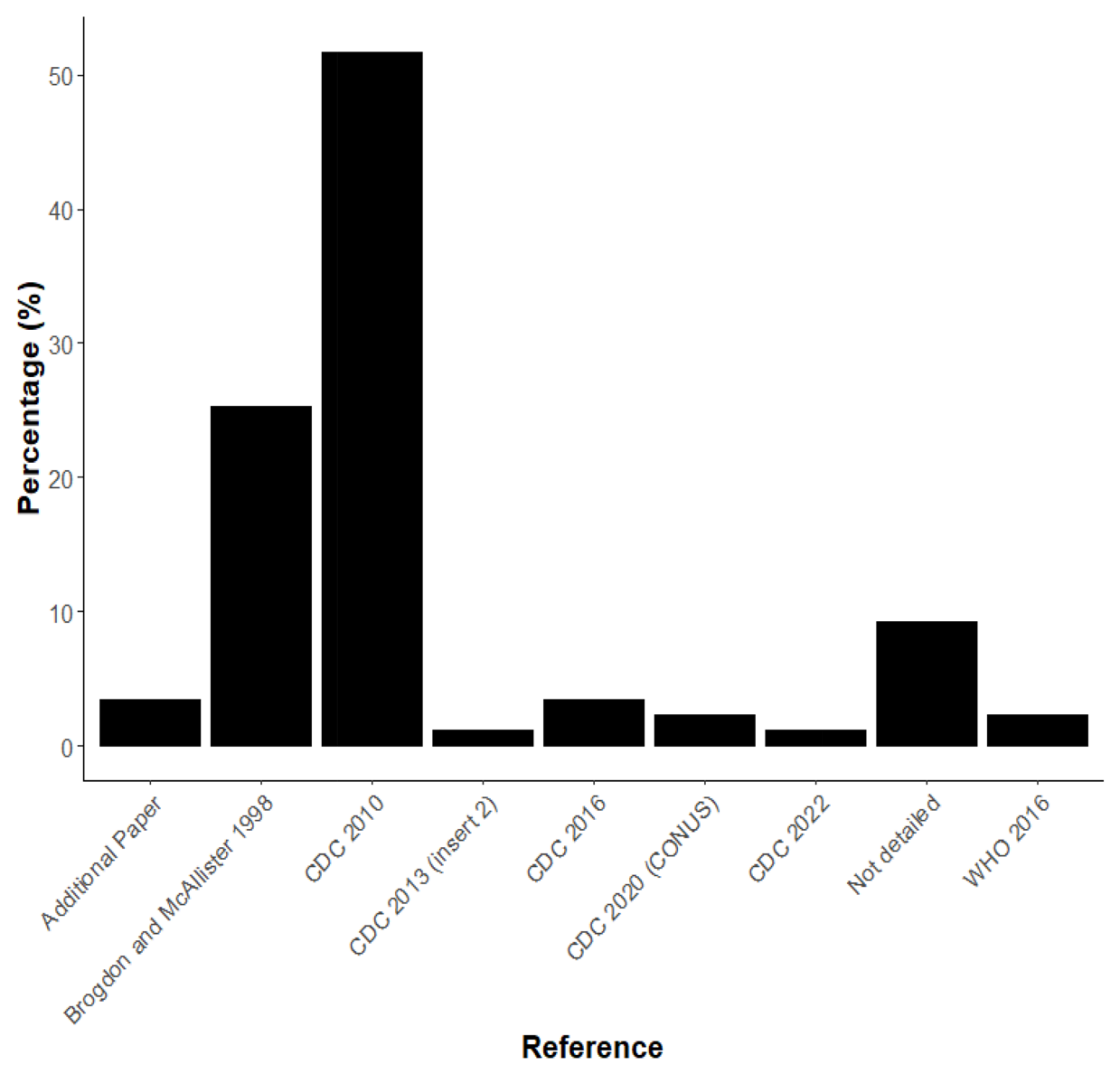
Percentage of guidelines referenced in publications.

Of the papers reviewed, there were some inconsistencies with the way that published methods were cited. Most papers referenced ‘CDC (2010)’ “Guidelines for Evaluating Insecticide Resistance in Vectors Using the CDC Bottle Bioassay” (52%) ^8^. This was also referred to as ‘Brogdon and Chan (2010)’, with a range of incorrect authors and publication years referenced including: Brogdon and Chan: 2012, 2013 and CDC: 2011, 2012, 2013 and 2019 being given for this 2010 reference. No papers referenced the updated CDC (2023) “Manual for Evaluating Insecticide Resistance Using the CDC Bottle Bioassay” ^5^. Brogdon and McAllister (1998) were referenced in 25% of papers, despite this not being a clear instruction document and 9% of studies did not give a reference for the methodology they used ^3^ ( Figure 2).

**Figure 3.**
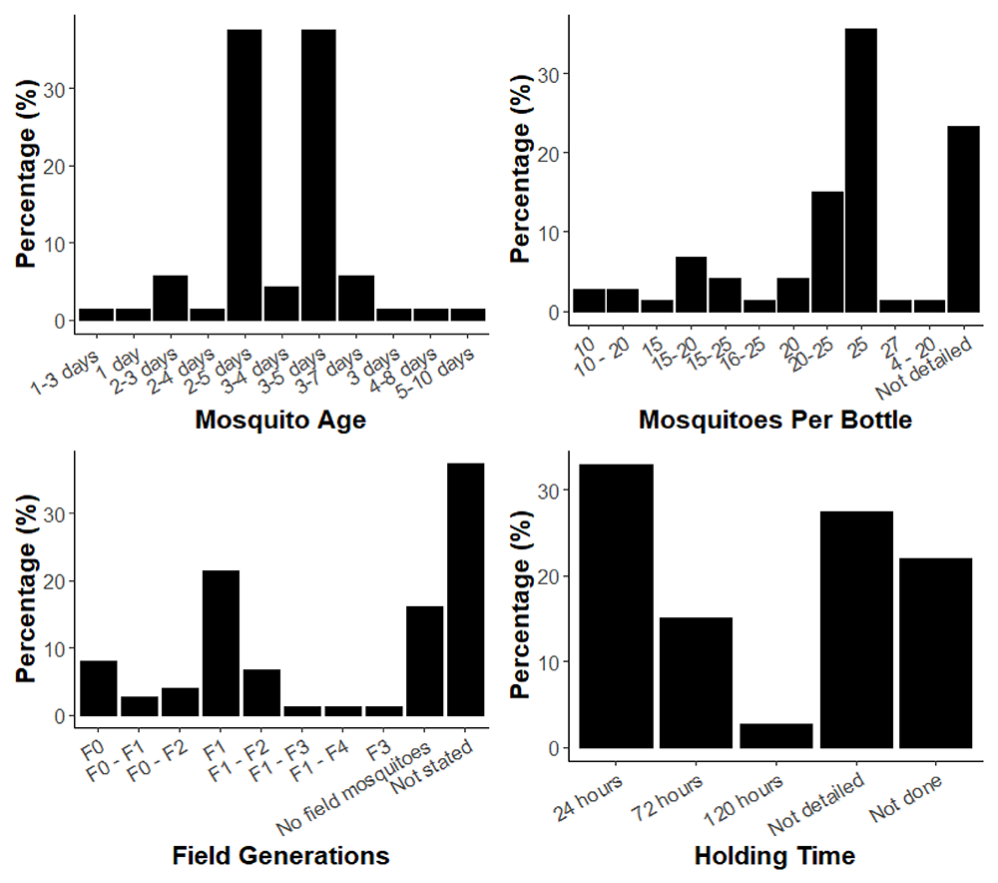
Graphs of the age range of mosquitoes reported in the reviewed publications ( **A**), the number of mosquitoes per bottle reported in the reviewed publications ( **B**), the time post-collection of mosquitoes used in studies which used field-derived mosquitoes ( **C**) and holding time of mosquitoes post-exposure to insecticide ( **D**).

The reported methodologies in the reviewed papers did not always match the published guidelines, which may partly be a result of the fact that parameters have changed over time. Firstly, recommendations for the age of mosquitoes to be used in the bottle bioassay has changed between iterations of the published guidance. The original method published by Brogdon and McAllister (1998) recommended a testing age range of 3–4 days, no age was specified in the CDC (2010), and the updated CDC (2023) manual recommends 2–5 days ^3, 5, 8^. Most studies reported having tested with mosquitoes within an age range of 2–5- or 3–5-days, together accounting for 76% of the total papers ( Figure 3A). Only 4% of studies reported testing with 3–4-day old mosquitoes. 8% of studies reported using mosquitoes older than 5 days and 2% less than 2 days old. The published protocol for WHO 2022) specifies an age range of 3–5 days, yet one paper referenced this document and used mosquitoes aged 2–5-days ^18^.

The reported number of mosquitoes per bottle was also variable ( Figure 3B). The CDC (2010) and CDC (2023) guidance procedures do not include specific recommendations for the number of mosquitoes per bottle but do suggest testing 100 per treatment over 4 replicate bottles and more specifically that 10–25 mosquitoes are required per control ^5, 8^. 74% of studies reported using 10–25 mosquitoes. This is a broad range however, and the number of mosquitoes per bottle could potentially impact mortality outcomes by altering the level of contact with the insecticide treated surface as seen with the WHO tube bioassay ^12^. In contrast, the WHO (2022) recommends testing as close to 25 mosquitoes per bottle as possible to avoid crowding; 36% of all the reviewed studies reported using this number, whether for the CDC or WHO bottle bioassay ^18^. 32% of studies reported a range of mosquitoes tested per bottle and not a specific number which would allow an accurate ‘n’ number per treatment to be calculated. 23% of studies did not report the number of mosquitoes tested per bottle at all ( Figure 3B). The Brogdon and McAllister (1998) method states that 5 or fewer mosquitoes can be tested; only a small number of studies (3%) reported using 10 or fewer mosquitoes per bottle ^3^. In contrast, the WHO (2022) recommends testing as close to 25 mosquitoes per bottle as possible to avoid crowding; 36% of all the reviewed studies reported using this number, whether for the CDC or WHO bottle bioassay ^17^. The CDC and WHO bottle bioassays are both principally used to monitor for the emergence of resistance in field populations, and in this situation the mosquitoes used for testing may be directly caught in the field (F0), or reared for a generation or more in the lab to produce sufficient mosquitoes for testing (F1 denotes the progeny of wild caught mosquitoes, F2 their progeny, and so on). 85% of papers used field-derived mosquitoes, commonly for purposes of detection of insecticide resistance and measurement of resistance intensity within countries and geographical regions to inform appropriate vector control interventions. Additional purposes include using the bottle bioassay to determine resistance status followed by further molecular exploration of specific resistance mechanisms, for example the use of synergist assays to determine the presence or absence of metabolic resistance mechanisms. A range of generations of field mosquitoes were tested in the studies, from F _0_–F _4_ ( Figure 3C). It had not been specified in any of the guidelines which generation of field-derived mosquitoes to test with until the CDC (2023) guidelines, which recommend testing only up to the F _2_ generation. This may explain why 44% of studies which tested with field-derived mosquitoes did not specify the generation used. A total of 20% of studies also reported using a range rather than a specific generation. The CDC (2010) and CDC (2023) guidelines advise to test multiple bioassays over several days and pool the results to achieve the desired sample size, which could explain why a range of generations was tested in these cases ^5, 8^.

There was consistency in how the amount of insecticide used to coat bottles was reported in the reviewed papers, with dose or concentration reported in µg/bottle or µg/mL; since 1mL of insecticide solution is used to coat bottles these units are equivalent. Of the 24% of papers that tested with a synergist, most of them used the recommended CDC guidance concentrations for S.S.S-tributlyphosphorotrithioate (DEF) at 125µg/bottle, diethyl maleate (DM) at 80µg/bottle and ethacrynic acid (EA) at 80µg/bottle. For Piperonyl butoxide (PBO), the most used synergist, 44% of papers used the CDC (2010) recommended concentration of 400µg/bottle ^8^. 13% of papers used the lower concentration of 100µg/bottle recommended in the updated guidelines CDC (2023) however all pre-dated this revised guidance ^5^. The WHO recommends the use of the WHO tube bioassay and not the WHO bottle bioassay for PBO exposure ^7^.

Finally, a substantial percentage of the studies either did not detail a holding time post bioassay (27%) or did not carry it out as part of the methodology (22%), as shown in Figure 3D. The CDC (2010) and CDC (2023) guidelines do not suggest a recommended holding time for mosquitoes post-exposure ^5, 8^. The WHO (2022) guidelines recommend 24 hours for pyrethroids, neonicotinoids including clothianidin, and butenolides and 72 hours for chlorfenapyr tested in WHO bottle bioassays ^7^. The WHO (2022) guidelines for the tube test recommend 24 hours holding time which could explain why 33% of studies used this time point as it is a recognised measurement for mortality for bioassays outside of the CDC bottle ^18, 35^.

### Experimental investigation: understanding the level and sources of variability in results from the WHO bottle bioassay

**Figure 4.**
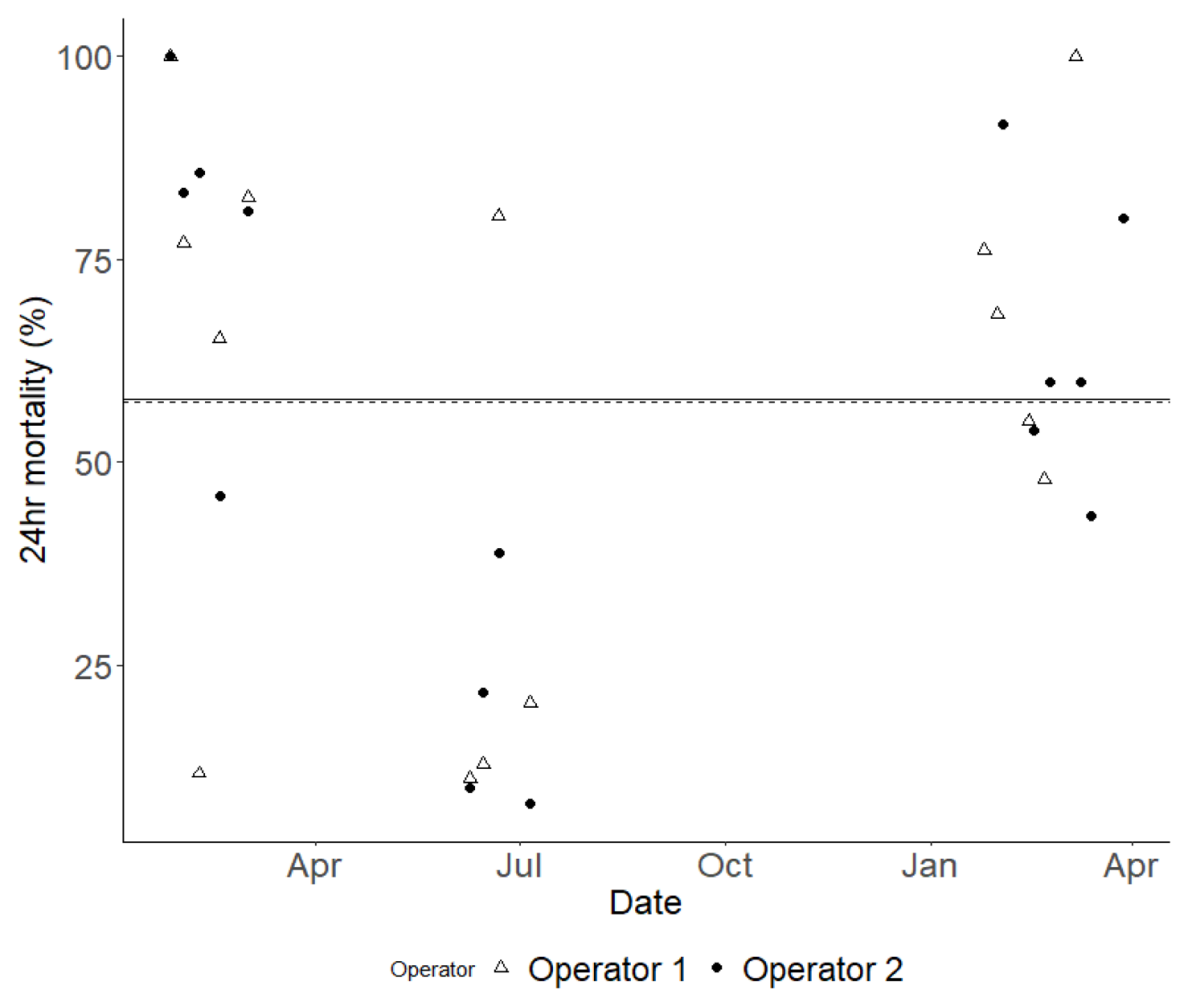
The variation in mortality rates 24 hours after exposure was observed throughout the study, comparing the results from two different operators. Each data point indicates the outcome of a single bioassay, with Operator 1's results marked with open white triangles and Operator 2's marked with solid black dots. To illustrate the average mortality rate for each operator over time, a dashed line represents Operator 1, while a solid line represents Operator 2. It is important to note that these average mortality lines nearly overlap.

**Figure 5.**
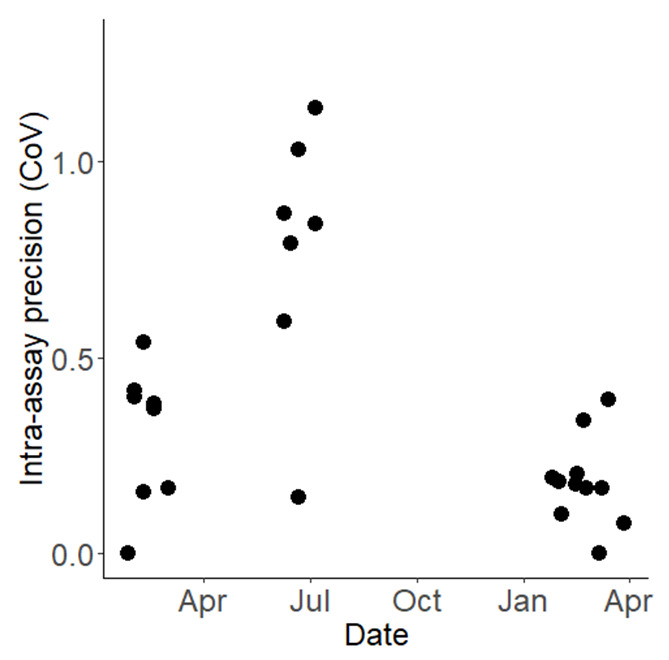
Intra-assay precision (Coefficient of Variation, CoV) for the WHO bottle bioassays conducted between February 2022 and April 2023. Each point on the graph denotes the precision of an individual bioassay, plotted over the course of the testing period.

To characterise and quantify the inherent variability in results from WHO bottle bioassays, 9,130 susceptible Kisumu mosquitoes were exposed to the same concentration of permethrin, systematically distributed across 365 bottles. The mean mortality across all 365 bottles was 67.18%, however, the confidence interval was wide, ranging from 38.29% to 84.47% ( Figure 4). The overall Coefficient of Variation (CoV) for the WHO bottle bioassay across the study was 0.682. This overall variability is attributable primarily to variation between testing days rather than within them: the standard deviation between days was 1.788 (equivalent to a variance of 3.200, Figure 5) while the within-day standard deviation was just 0.145 (equivalent to a variance of 0.021). If the summer period of 2022 (where mortality was unexpectedly exceptionally low and the CoV was markedly higher, Figure 5) is excluded, then the overall CoV declines to 0.414.

**Figure 6.**
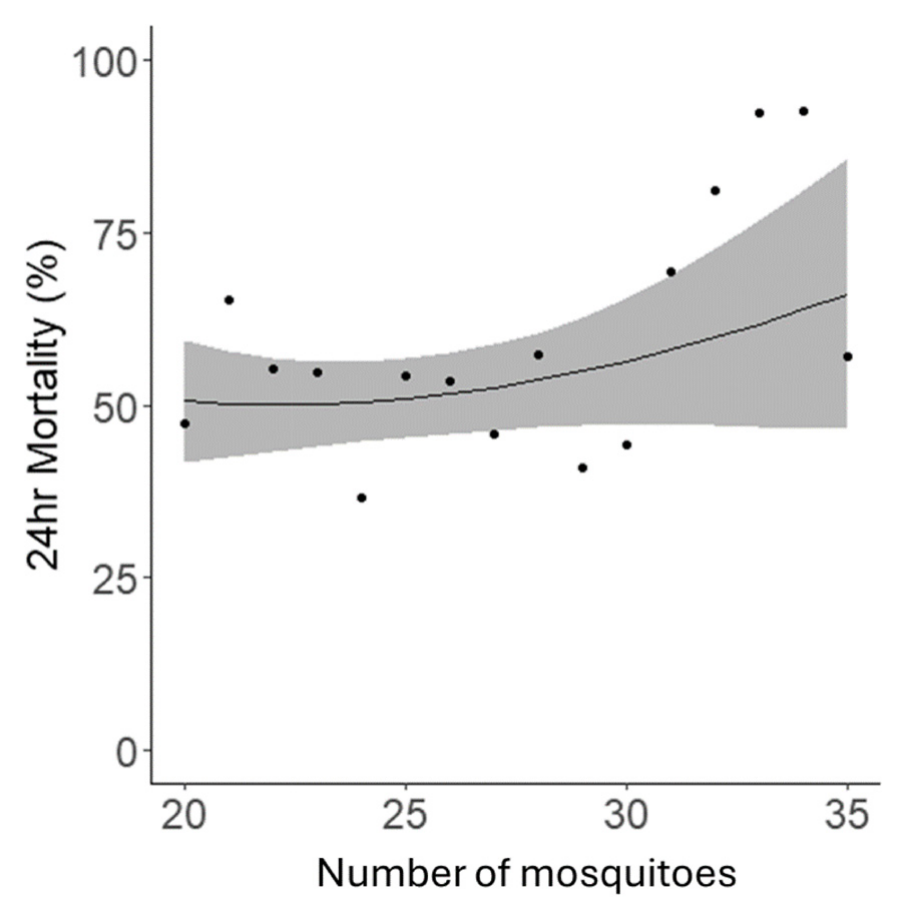
The relationship between the number of mosquitoes per bottle in a bioassay and mortality rates 24 hours post-exposure. Data points indicate the mean mortality for all tubes with that specific number of mosquitoes. The black line represents the fitted trendline, and the blue shaded area depicts the 95% confidence intervals for this model.

Whilst the protocol stated that 25 mosquitoes should be exposed per bottle, due to the challenges of handling live mosquitoes the precise number exposed in each bottle varied, though all bottles contained between 20–35 mosquitoes, as per the protocol’s specification. Within this range, the number of mosquitoes per bottle had no statistical relationship with 24hr mortality (p=0.18) ( Figure 6).

**Figure 7.**
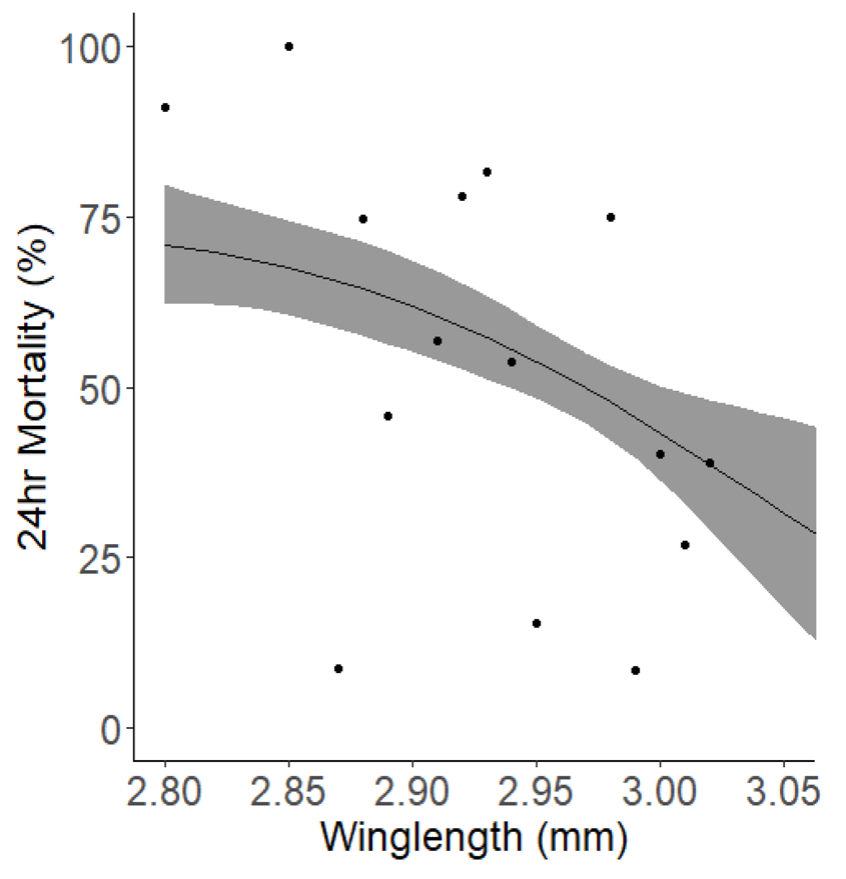
Relationship between wing length and mortality 24-hour post-exposure. The solid black line indicates the best fit trendline from the model, while the surrounding grey shaded area denotes the 95% confidence interval for the trend. Each point represents the average mortality rate observed for each wing length (grouped to the nearest) 0.01 mm.

Looking into other parameters which may affect mortality in a WHO bottle bioassay identified mosquito dry weight and relative humidity (RH) as having a significant impact. The data suggested that RH at the start of exposure was a marginally stronger predictor compared to RH at the end of exposure. However, they have similar predictive capacities which was minute. When we integrated the additional parameter of wing length, an alternative measure of mosquito size, into our analysis, the significant association between RH and mortality ceased to be significant, either at the beginning (p=0.531) or the end (p=0.332) of exposure. Wing length showed a general negative correlation with mortality ( Figure 7).

## Discussion

We performed a multifaceted study to enhance our understanding of the CDC and WHO bottle bioassay methods, common methods used to collect data on mosquito susceptibility to insecticides. Our research involved a) analysing published guidance to determine how specifics of the methodological details have changed through historical iterations, b) reviewing literature for bottle bioassay studies to understand the variation in the methods used and how they are reported, c) attempting to compare results between studies which reported testing results from the same combinations of mosquito strain and insecticide, and finally d) by conducting laboratory controlled experiments. The primary objectives of the experiments were to identify key experimental variables and understand the impact of changing parameters on the entomological endpoints of interest. This approach is valuable because the effects of these parameters on results are not well-understood, making it difficult to compare and interpret data from different studies effectively.

Reviewing published literature for studies which reported results from bottle bioassay experiments, either CDC or WHO bottle bioassays, identified 76 papers. Among these publications there was a lot of variability in the level of detail reported about the methods used for testing, but the general trend was for the specific methodology not to be reported in much detail at all. For 1 in 10 publications guidelines were not cited at all for these standard protocols. In many cases authors report not the actual method used, but rather quote the published guidance, for example reporting that ’20–30 mosquitoes’ were exposed per bottle as per the cited guidelines rather than reporting the real ‘n’. When details of testing parameters such as mosquito age or the number of mosquitoes exposed per bottle were reported the values varied between publications and even between the reported methods and the guidelines cited in the paper. The fact that guidelines have evolved, and specific details changed over time no doubt contributes to this confusion, as might the fact that some elements of the available guidelines are open to interpretation or details are not specified. Additionally, the more recent WHO 2022 guidelines are only recently published and may not have been taken up immediately across research groups, so this would explain why these guidelines are less referenced. For example, Brogdon and McAllister ^3^ describe the CDC bottle assay method with 25 mosquitoes per bottle. The WHO tube bioassay is then cited as more practical as it allows for testing with as few as 5 mosquitoes.

Variability in reported methodological detail makes it difficult to compare or contrast results between published studies, even when the testing method used is the same. In this case the only studies which tested the same strain and insecticide combination and could be compared all reported over 90% mortality, unsurprising since this data consisted of insecticide-susceptible lab populations exposed to discriminating doses designed to identify insecticide-resistance. From this review it was therefore difficult to determine whether discrepancies in methodology interpretation between studies was influencing the mortality data generated when using this bioassay. Reporting of more granular discriminating times generated through analysis of time-response data would facilitate comparison between studies and strains.

Even when precisely the same method is used to test the same insecticide against the same mosquito population under consistent testing conditions, a bioassay will have some level of inherent variability. Our investigation set out to quantify the WHO bottle bioassay method’s variability under controlled conditions, yielding significant insights for understanding and conducting these bioassays. A previous study showed that variability within World Health Organization (WHO) bottle bioassays was consistent for specific insecticides like chlorfenapyr and clothianidin across various mosquito species. However, for other insecticides such as flupyradifurone and transfluthrin, the variability significantly increased. Notably, with flupyradifurone, the variability in mortality rates for *Ae. aegypti* was as high as 12%, compared to other insecticides where the mean variability remained below 10%. The study also highlighted that pyriproxyfen exhibited a within-assay variability of 2.5% for *An. gambiae s.s*. and 3.4% for *An. stephensi*, highlighting differences in response to insecticides among species ^20^. As a result, it is critical to understand the inherent variability in bioassay results to perform meaningful power calculations and then to interpret results properly, i.e. determining what is a real difference between treatments or populations and what might be noise. It is also interesting to compare the level of variability between bioassays to help in selection of the appropriate method. Even before the planned experiments were performed, we found evidence of variability in results between studies or over time – the LC _50_ previously established in the same laboratory with the same protocol for the same mosquito strain did not hold true at the time of this study. This may be due to changes in rearing conditions over time, test material variability such as the insecticidal stock, which was previously synthesised by the manufacturer with higher purity, and although test concentrations remained the same its possible additional impurities impacted biological efficacy, or between cohorts of mosquitoes reared at different times or variability or ‘noise’ in data affecting the result returned by the log probit analysis of dose response data. Having re-established an LC _50_ we then controlled as many variables as possible in testing 9,130 mosquitoes across 365 bottles. Our results revealed an average mortality rate of 67.18%, with a confidence interval ranging from 38.29% to 84.47%. This study is notable for its scale compared to similar studies in existing literature, which often involve lab-reared mosquito colonies and the same insecticide in bottle bioassays. Importantly, to our knowledge, this is the first study of its kind to consistently control and monitor a range of factors that could influence the results over such an extended period within a single lab. Replicates where control mortality exceeded 20% were excluded from the analysis, and control mortality in the analysed data ranged between 0 and 20%. This decision underscores the nuanced understanding that, while mean control mortality provides a baseline for comparison, the variability within these controls—ranging theoretically from homogeneity to significant heterogeneity—can profoundly impact the interpretation of experimental outcomes. Such variability gives some insight into the potential influence of operator handling and experimental conditions, emphasizing the importance of examining not only mean mortality but also the distribution of outcomes across replicates to ensure the reliability and reproducibility of our findings.

Exploring the variability and excluding other explanatory variables, we observed a within-day variance that corresponds to a standard deviation of 0.331 (numeric value: 0.109). In contrast, the between-day variance was more pronounced, with a standard deviation pegged at 2.069 (numeric value: 4.282). Furthermore, the data underscores a significant aspect of long run precision, characterized by a Coefficient of Variation (CoV) standing at 0.69. It is essential to design studies with enough statistical power and relevant temporal spread to consider variations in data. Doing so would enable a more nuanced approach to detection of any changes in resistance, allowing for better-informed decisions in monitoring and managing resistance.

With regards to precision, our analyses showed that the inter-assay and intra-assay precision of the bottle bioassay method was much higher than the WHO tube method (unpublished data). This might be partially explained by the fact that the bottle method was less impacted by the number of mosquitoes per bottle, even though it was more susceptible to relative humidity changes. This difference in intra assay precision was reflected in lower mortality and higher mosquito dry weights. Though standard mosquito counts were used for bioassays conducted during this period. One specific observation from assays conducted in 2022 was a minor disparity in the mosquito count between bioassays set up by each of the two operators. On average, both operators aspirated around 25 mosquitoes per bottle. However, Operator 1 tended to aspirate slightly more than this average, whereas Operator 2 showed a tendency to aspirate slightly less. This trend, however, did not extend into the 2023 assays. In analysing the number of mosquitoes per bottle, there was a trend towards increased mortality with the highest numbers of mosquitoes tested, and a general positive correlation. Though this trend was not statistically significant it is supported by a previous study which demonstrated that the number of mosquitoes per test unit influences mortality and knockdown rates, with higher numbers resulting in greater insecticide exposure and effectiveness ^12^. However, it should be noted that upon further analysis with additional variables like wing length accounted for, the previously significant association of relative humidity with mortality disappeared (either as measured at the start (p=0.531) or the end (p=0.332) of the exposure period).

While initially differences were noticed there were no significant time or operator trends observed once core variables were accounted for, indicating that these bioassays were consistent in terms of these factors. Operator differences are not accounted for in the current guidance however it is encouraging to see that at least under controlled conditions of this study that this did not significantly impact the bioassay outcomes - this is a highly controlled study and so this may not be a surprise. We did observe a substantial difference between different 'experimental periods,' which highlighted the need for careful consideration of temporal factors in planning and interpreting these bioassays. Mosquitoes reared for the bioassays we conducted in the summer of 2022 were larger measured as dry weight than other testing periods, and wing lengths were on average about 10% bigger. Our modelling indicated that this was a key predictor of reduced mortality, though our analysis would only predict mortality to be about 5–10% lower and in fact the mortality showed a significant 52% drop from 73%±5% in Spring 2022 to 20%±4% in Summer 2023 before returning to 67%%±5% in Spring 2023. Additionally control mortality during this period was also lower. It is unclear why the mosquitoes during this period showed such an increase in size, but it could be due to subtle differences in rearing conditions.

By controlling as many variables as possible, we were able to examine the effect of changes in some parameters which varied beyond our control, to find those which were most influential, and which should therefore be controlled as far as possible in standardised protocols. Where they cannot be controlled, they should be recorded and used in interpreting the test data. By analysing the data and using the meta-data generated in this study, we were able to examine the effect of four parameters on observed mortality: testing conditions (temperature and relative humidity), age of mosquitoes, number of mosquitoes exposed per bottle and size of mosquito.

There is a large body of evidence of the impact of temperature on mosquito however relative humidity less often directly examined. Multiple *Anopheles* mosquito species have been shown to be less susceptible to pyrethroids at higher temperatures ^25^, which could be attributed to enhanced enzymatic activity in the mosquitoes at elevated temperatures leading to an increased rate of insecticide detoxification. Temperatures below the recommended 27 ± 2°C have been seen to influence the tolerance of *An. arabiensis* and *An. funestus* to both bendiocarb and deltamethrin ^36^. In a Ugandan field insectary without environmental controls, but with monitored temperature and humidity, there was a pronounced and statistically significant decrease in the mortality of *A. gambiae* mosquitoes as humidity levels rose ^37^, though others have seen a high degree of tolerance to a wide range of relative humidity (RH) levels in *Anopheles*, with their survival not significantly impacted between 60% to 100% RH ^14^, though RH levels below 10% prove to be lethal within hours. Some studies suggest that mosquitoes, especially those from arid regions, might develop resistance to desiccation, potentially surviving low relative humidity conditions slightly longer than laboratory-bred counterparts ^38^. Even if relative humidity has a limited impact on bioassay outcomes it is plausible that a reduction in longevity due to low relative humidity would compound the effects of bioassay mortality and mosquitoes may appear less resistant to insecticides. Temperature and RH were not reported in the published studies we reviewed, and so the recent addition of a RH range to the new CDC guidance for the bottle bioassay method is welcome ^5^. In situations where environmental conditions cannot be fully controlled, it is crucial to report the environmental conditions in detail to ensure that the data can be correctly interpreted.

In the published literature most studies reported testing with mosquitoes 2–5- or 3–5-day old. The recommendation of 2–5-days was introduced in the CDC 2023 guidance; however, this age range was potentially commonly used prior to this due to guidelines already in place for the WHO cone test and the WHO tube bioassay ^18, 35^. Studies which reported mosquitoes in the testing age range of 3–5-days all dated from 2015 onwards. WHO 2013 guidance was published 2 years prior to this recommending this age range, which could explain this shift. The wider age range category used in the CDC guidance could be due to the challenges of collecting field mosquitoes within a small window of time. Mosquito age has been shown to have an impact on resistance status, with larvae and adults that are older having increased susceptibility ^39^. It is therefore important to have consistency between published guidelines to ensure standardisation of testing ages and comparability of data.

In terms of the number of mosquitoes per bottle used in the bioassays, insecticide delivery relies on mosquitoes contacting the treated surface of the bottle and the quantity of mosquitoes per bottle could influence resting and flight behaviour. An increase in flight behaviour could cause some mosquitoes to lose contact with the insecticide before receiving a lethal dose resulting in fluctuations in the mortality data generated. We found that there was no significant relationship with either knockdown or 24-hour mortality within the studied range of 20–30 mosquitoes, though a trend for reduced mortality below 20 and increased above 30. This possible reduction in mortality below 20 is in agreement with previous work which has shown a decrease in mortality in the WHO tube bioassay with number of mosquitoes ^12^. In the 2022 assays, there was a slight difference in the average number of mosquitoes added to each bottle by the two operators, with one operator tending to use numbers slightly above 25 and the second slightly lower, though in the 2023 assays no such difference was observed. The Brogdon and McAllister (1998) CDC bottle method states that 5 or fewer mosquitoes can be tested, but only a small number (3%) of studies we reviewed reported using 10 or fewer mosquitoes per bottle ^3^. Although no justification was provided for the low number, the mosquitoes tested were F _1_ field mosquitoes and obtaining sufficient field-derived mosquitoes can be challenging. Indeed, Brogdon and McAllister ^3^ suggest that results from multiple small-scale tests can be combined, though they do not provide data on the effect of numbers this low on mortality. More specific guidance on the quantity of mosquitoes exposed per bottle would help consistency between studies. As well as the quantity of mosquitoes per bottle, agitation of the bottle during testing could also have an impact on resting and flight behaviour. The published CDC guidance documents all suggest gently rotating the bottle while scoring mortality ^3, 5, 8^, though only 4% of published studies mentioned bottle rotation. Inconsistent agitation of bottles may create variability in results so either clearly specifying a suggested frequency for agitation or reporting methods accurately in studies would be beneficial.

Many studies have shown that mosquito body size is inversely related to insecticide susceptibility ^40^, often by artificially altering mosquito size by adjusting larval nutrition. Our study did not artificially adjust the mosquito size but instead allowed it to vary naturally. Although the size range is small because of standardised rearing protocols it still adds to this body of evidence on the impact of size on susceptibility ^27^. We measured both dry weight and wing length as a proxy for body size and found that with every 1 standard deviation increment in dry weight (from an average of 0.48mg to 0.54mg) there was an associated 13.35% reduction in mortality, and an increase in average of wing length from 2.94mm to 3.00mm led to a 4.54% reduction in mortality. Tight control of rearing conditions when generating cohorts of mosquitoes for testing is critical to ensure minimal variance in body size ^27^, and should ideally be monitored through a quality control process clearly defining minimum and maximum size thresholds for testing, using an appropriate size assessment methodology (be it wing length or dry weight) that is powered to detect significant differences in your test population over time. When testing field-derived mosquitoes it is important to record size alongside test results so that seasonal differences, for example, can be considered when interpreting results.

In addition to these four key parameters, there are some others which were not investigated during this study, but which may influence the measured endpoint. Physiological parameters of the mosquitoes other than size and age might be a source of variation and should be either kept consistent where possible or reported alongside data. All bioassays in this study were conducted during the same time period in the afternoon to minimize variability due to circadian differences in mosquitoes. However, time of day could influence mosquito activity and is a parameter worth investigating, particularly for insecticides with target sites or modes of action affected by metabolic activity of the mosquito. The current CDC 2023 guidelines specify testing no further than F _2_ generations in bottle bioassays ^5^, and CDC guidelines are to report the physiological status of the mosquitoes i.e., unfed, blood fed, semi-gravid, gravid on the results sheet.

As well as characteristics of the mosquitoes, testing parameters may also affect results. The CDC guidelines suggest that the orientation of bottles during exposure does not significantly impact results, provided that the method is consistently applied though we know of no experimental evidence. The orientation of a bottle during exposure could potentially influence the behaviour of mosquitoes within the bioassay, an idea supported by evidence from the WHO cone bioassay where angle of testing affects contact with a treated surface ^35, 41^. The World Health Organization (WHO) cone bioassay plays an integral role in the evaluation of the efficacy of long-lasting insecticidal nets as well as insecticides used in indoor residual spraying. The test is used on a variety of treated substrates, such as pieces of bed nets, mud, cement and wood. The cone setup assumes a wide variety of angles under different settings in which it is applied. However, the guidelines provided for the performance of the assay do not specify the angle at which the test must be performed, which could result in variations of flight behaviour within the assay and consequently insecticide uptake and mortality ^35, 41^. We are also unaware of any validation of show how evenly bottles are coated with insecticides using the methodology outlined in the CDC guidelines (2010 and 2023). It is possible that temperature and humidity could impact this drying process especially when surfactants are involved. Issues were seen with control mortality across sites within the WHO multicentre study when using RME ^19^. Mosquitoes show avoidance behaviour and irritancy to insecticides and mosquitoes may choose to rest in areas less coated with insecticide ^42, 43^ impacting insecticide uptake and in turn mortality. It would be interesting to explore behavioural resistance, mosquito behaviour that enables reduced contact with insecticides ^7^. The period which mosquitoes are held for after exposure for mortality scoring was not reported in all reviewed publications. Although the relevant holding time may be chemistry specific, standardising and reporting holding time is important to control for the mosquito recovery period, particularly in the case of metabolic resistance as the mosquito detoxifies the insecticide over time. The longer the holding period the greater the variability which might be introduced by factors such as desiccation of mosquitoes, or death of mosquitoes from spilling or drying out of sugar solution. 

## Conclusion

Upon reviewing the literature for publications reporting results of bottle bioassays there was general inconsistency in the precise methods used for testing and in the way that methodologies were reported and the relationship between reported methods and published guidance documents which were cited. Some publications incorrectly cited the CDC guidelines, an issue which could be overcome by a clear referencing format for the CDC 2023 guidance being made available. The specific methodology was often not reported in detail or the parameters which were documented varied between studies, making it impossible to compare results between published studies. These findings highlight the importance of having a standardised set of guidelines to follow for conducting the assay consistently and reporting. Since we have shown experimentally that changes to testing parameters can have an influence on the entomological endpoint, it is important that precise methodological detail is published with results, and with reference to published guidelines as appropriate.

Our experiments investigating the inherent variability in results from the WHO bottle bioassay method has underscored the significant impact of variables like mosquito dry weight, relative humidity, and mosquito count on the entomological endpoint (24h mortality). Notably, we found a substantial negative correlation between wing length and mortality, indicating that larger mosquitoes were less likely to die in the bioassay (p<0.001). This finding points to the necessity of rigorous quality control and standardized rearing practices for laboratory mosquito strains. Additionally, it emphasizes the need for detailed documentation of metadata related to mosquitoes collected from the field. We discovered that the dry weight of mosquitoes and relative humidity were core predictors of outcomes in the bioassay. In particular, the starting relative humidity was a slightly better predictor than the relative humidity at the end of the experiment, though both were almost interchangeable in their predictive power. Reports of experimental data should include specifics about mosquito collection, testing, and rearing methods, as well as information about the conditions under which bioassays are conducted, like temperature and humidity. Such measures will enhance the clarity and consistency of bioassay data interpretation. Our study demonstrates how minor variations in experimental parameters can introduce significant variability into bioassay outcomes, even under tightly controlled conditions. The validation of methods to characterise and generate a methods claim is an important part of being confident that the method selected to address a given question is appropriate and that the data that is generated is reliable. Part of a formal validation will include investigating the effect of altering testing parameters and characterising and minimising variability ^44^. Where relevant, generation of consensus SOPs which are developed and agreed by key practitioners ^45^ can help with the uptake of standardised methods which aid interpretation of results between studies.

The inclusion of technical and biological replication in resistance monitoring testing is vital for detecting and accounting for these variances in bioassay results, so that data can be interpreted meaningfully, strengthening evidence-based decision making in vector control. Currently CDC guideline recommendations of 100 per test concentration does not seem sufficient considering this level of variation in this method and it is possible that this guidance needs updating ^5^. Another factor that should not be ignored are the differences in endpoint which are observed between testing days. Our analysis of data collected over repeated testing sessions highlights the importance of spreading testing out over more than a single day to be able to get a more representative data set and allow you to be more confident in your conclusion about the presence or absence of resistance in a mosquito population. Currently there is no guidance around spreading testing over numerous days, but this kind of could allow for smaller tests to be conducted over multiple days which could be more feasible in settings where large numbers of testing age mosquitoes are not necessarily available on the same day. We recognise that we only studied variability and testing parameters with a pyrethroid, and interesting further work might be to repeat elements of this study with other insecticides either using currently used pyrethroid insecticides such as alpha-cypermethrin or deltamethrin, or insecticides from other classes such as chlorfenapyr or clothianidin for which susceptibility is also assessed using bottle bioassay.

Generating more robust and consistent susceptibility data using the bottle bioassay is possible through tightening up the methodology to minimise variability and recording meta data alongside bioassay results to allow more informed interpretation. More accurate reporting of the precise methods used to generate bioassay data will facilitate better comparability of data sets, which should include reference to relevant published guidelines and reporting of raw data and actual rather than target values for parameters such as number of mosquitoes exposed per bottle or temperature in the testing room. Generation and reporting of better data in these ways will facilitate better decision-making in designing vector control strategies, contributing to more effective efforts to combat mosquito-borne diseases.

## Data Availability

The bibliographic references used for the literature review are included in the References section. All raw data contributing to the results presented in this paper are freely available in an online repository. Datasets are readily available at: https://github.com/i2i-Data-Repository/WHO-and-CDC-Bottle-Bioassay-Paper-2024/tree/main Repository: Unpacking WHO and CDC Bottle Bioassay Methods: A Comprehensive Literature Review and Protocol Analysis Revealing Key Outcome Predictors URL: https://github.com/i2i-Data-Repository/WHO-and-CDC-Bottle-Bioassay-Paper-2024/tree/main Noise_Bottle_AllTimepoints.csv Data sets and r scripts: 1.Noise_Bottle_Dose_Response.csv – data for preliminary dose response to identify LC50, used for the main experiments. 2.Noise_Bottle_Main_Experiments.csv - knockdown and mortality data for the main experiment, includes environmental conditions and mosquito body size indices. 3. Noise_bottle_Script.R - r script used to analyse the data and produce publication plots. Data are available under the terms of the Creative Commons Zero "No rights reserved" data waiver (CC0 1.0 Public domain dedication).

## Software availability

Source code available from: https://github.com/i2i-Data-Repository/WHO-and-CDC-Bottle-Bioassay-Paper-2024/tree/main.
