# Supplementary material for: Unpacking WHO and CDC Bottle Bioassay Methods: A Comprehensive Literature Review and Protocol Analysis Revealing Key Outcome Predictors
Source: Gates Open Res. 2024 Dec 18;8:56. Originally published 2024 Jun 4. [Version 2] doi: 10.12688/gatesopenres.15433.2 (PMC11335745; doi:10.12688/gatesopenres.15433.2)
Supplement: Peer review history

# Reviewer report: Reviewer response for version 1

**DOI:** 10.21956/gatesopenres.16785.r37721

**Published:** 2024-09-05

**Authors:** Theeraphap Chareonviriyaphap

**Affiliations:** 1 Kasetsart University, Bangkok, Thailand

Assessment of mosquito insecticide resistance using bottle bioassay has various guidelines launched in the past decades. Each guideline has some different procedures, which might affect data interpretation. Therefore, this article aimed to 1) summarize all guidelines, either CDC or WHO, 2) review variation of previous articles, and conduct the experiment to observe variation caused during the experimental study.

 Overall, the idea of this article is great and informative, as it combines up-to-date guidelines for readers and highlights all potential variations that might influence the outcome. However, there are several minor flaws in this article: 1) no rationale is provided for the selection of insecticides for the experimental study, 2) no statistical analysis was performed using GLMMs as mentioned in the data analysis, 3) the structure of the methods section should be laid out to facilitate reader comprehension, and 4) the discussion and conclusion sections are overly lengthy. Additionally, I have more suggestions listed below:

**Introduction**

1. Please check whether the WHO tube bioassay protocol mentions 1 h knockdown or mortality and insert reference:
  “It was developed as an additional means of monitoring resistance to the original World Health Organization (WHO) tube bioassay, which measures mortality 1- and 24-hours post-exposure”
2. Please change mosquito to mosquitoes
  “Data generated using the assay is widely used for the routine monitoring of resistance to commonly used insecticides in field populations and the characterization of laboratory populations of mosquito”
3. High or low temperatures that increase insecticide-induced mortality
  “Humidity has been shown to potentially increase insecticide-induced mortality in mosquitoes”
4. The last two paragraphs should be combined and rewritten to shorten and more precisely lead to the aim of this article.

**Methods**

1. What is the rationale for ordering guidelines? Is it better to organize according to chronological order?
2. I am not sure whether my understanding is right that you would like to infer “ **this**” to the WHO bottle assay. If yes, I think you should change it or make it clearer to the reader:
  “This method is an adaptation to the CDC bottle bioassay where time to kill scoring is no longer done”
3. This paragraph should be moved to the result section:
  “An outline of the differences between these methods documents is detailed in……………”
4. The paragraph below should be shortened:
  “Studies where the MCD bottle bioassay was used……………..”
5. Methodological parameters in **Table 1** should be grouped and detailed in separate rows, categorized by aspects such as mosquitoes, insecticides & reagents, techniques, etc.
6. Please check in the literature review subsection, the final article is 74 (in text) or 76 (figure 1)?
7. The diagram representing article selection should be modified according to the descriptive data in the text, which will help the reader to understand at first glance. The current version is difficult to understand and follow.
8. How many papers?
  “Papers were removed which had been included in the previous searches”
9. Change Anopheles, Aedes, and Culex to italic
  “it is a novel method which is likely to be widely adopted for susceptibility monitoring in Anopheles, Aedes, and Culex mosquitoes”
10. Is it necessary to include **table 2**?  I think all the information was mentioned in the text about conditions and parameters that the author is interested in. If the author would like to keep it.
11. I did not see the rationale why you selected to observe 4 parameters in this study.
12. This sentence was repeated twice:
  “Insectary conditions were maintained at 26 ± 2 °C and 80 ± 5% relative humidity (RH), with L12:D12 hour light: dark cycle and a 1-h dawn and dusk. The larvae were reared in purified water and fed ground TetraMin® tropical flakes (Blacksburg, VA, USA).”
13. Why did you keep species of mosquitoes in the last paragraph? I propose to mention it in the first paragraph.
14. Please clarify what is phases1 and 2
  “Two phases of bioassay testing were done, with the methodology for phase 2 adapted phased on preliminary findings from phase 1:………………….”
15. I noticed that 20-30 mosquitoes were used in the experiment, but the result section showed a range of mosquitoes from 20-35 individuals. Could you please recheck it?
16. What is the purpose to include this sentence:
  “For every 5th or 15th minute can be accessed and retrieved from the system. During both testing and holding periods throughout the full run of the experiment, the temperature and humidity did not fall outside of the applicable control limits”

**Results**

1. It is good to use GLMMs, however I did not see results from this analysis. Could you please show and draw out what is the main contribution to the variation of outcome?
2. The results from this experiment showed mortality in control between 5-20%, however no Abbott's correction was performed. Could you please adjust and reanalyze?
3. These sentences were repeated twice:
  “At the time this new WHO methodology had several drawbacks: the test kit was seen to be expensive; test papers were not provided by WHO for certain insecticides; there was no provision for assessment of metabolic resistance; and the recommended diagnostic doses were not appropriate for all species. As a result, CDC looked to develop a bioassay methodology to address these issues29”
4. Please consider changing to a citation instead of a text
  “published in the Journal of the American Mosquito Control Association in 1998.”
5. Please insert ref in this paragraph properly:
  “Two additional documents were published to supplement the CDC (2010) guidelines…………”
6. As the article is overly lengthy, could you please consider minimizing or deleting large portions of text that repeatedly mention information from **Table 1**? Since all the information is already included in the table, there's no need to describe it again in the text.
7. In the literature review section, could you please indicate the number of publications followed by the percentage in parentheses for each subsection? This would be great for understanding.
8. In the literature review section, the percentages provided in the text indicate some missing data. Could you please ensure that all 100% are accounted for?
9. **In Figure 3**, please insert letters A, B, C, and D in each subpicture in correlation with descriptive information.
10. Comparing citations or popularity between the CDC guidelines (first published in 1998 with subsequent updates) and the WHO guidelines (published in 2022) might be misleading to readers. No publications have cited the WHO guidelines because it was recently published, making it difficult to follow the new procedures and publish within one year as the inclusion criteria (search article up to 2023) in your article.
11. Please consider subsection holding time and discuss why the previous article performed a holding period at a different time.
12.  Why is the rational to combine RH and wing length? Do you think both variations link to each other? If yes, please give some sentences before your result
  “When we integrated the additional parameter of wing length, an alternative measure of mosquito size, into our analysis, the significant association between RH and mortality ceased to be significant, either at the beginning (p=0.531) or the end (p=0.332) of exposure.”
13. **Figure 7**, change winglength to wing length

**Discussion**

1. Please consider moving this information to previous paragraphs mentioning about generation of mosquitoes:
  “The current CDC 2023 guidelines specify testing no further than F2 generations in bottle bioassays, and CDC guidelines are to report the physiological status of the mosquitoes i.e., unfed, blood-fed, semi-gravid, gravid on the results sheet.”
2. When comparing the CDC and cone tests, you suggest that the angle of the CDC bottle affects mortality. Are you confident about this statement? Do you have any scientific evidence? The cone test only involves impregnated fabric coated with insecticide, while the cone compartment itself does not contain any insecticide. In contrast, the bottle bioassay coats all parts of the bottle with insecticide. Thus, angle change affects to resting behavior of mosquitoes in the contest, but what about bottle bioassay? Do you have any other hypothesis?
3. Please consider shortening the discussion and conclusion sections.
4. Please include up-to-date research articles that conduct CDC bottle bioassay.

**References**

1. Ref 14: change *s.s. *to s.s.
2. Ref 30: change Anopheles albimanus to *Anopheles albimanus*

Is the work clearly and accurately presented and does it cite the current literature?

Yes

If applicable, is the statistical analysis and its interpretation appropriate?

Partly

Are all the source data underlying the results available to ensure full reproducibility?

Yes

Is the study design appropriate and is the work technically sound?

Yes

Are the conclusions drawn adequately supported by the results?

Yes

Are sufficient details of methods and analysis provided to allow replication by others?

Yes

Reviewer Expertise:

Vector biology and control

I confirm that I have read this submission and believe that I have an appropriate level of expertise to confirm that it is of an acceptable scientific standard, however I have significant reservations, as outlined above.

# Response

**Published:** 2024-12-16

**Authors:** Rosemary Lees

**Affiliations:** Liverpool School of Tropical Medicine, UK

We thank the reviewer for engaging so thoroughly with the article, and for making these suggestions, most of which we have incorporated into version 2 and believe it is improved as a result. We would like to address some specific comments below.

 Regarding the the rationale for ordering guidelines in the Methods section, the guidelines are in chronological order, apart from the WHO guidelines at the end, a sentence is included to explain this: "Additionally, the guidelines which outline the new WHO bottle bioassay method were considered:"

 The paragraph starting “An outline of the differences between these methods documents is detailed in……………” describes a table which highlights the difference in parameters between guidelines, with the aim of justifying the purpose of the study, and we therefore think it belongs in the Methods and not the Results section.

 The diagram representing article selection has been reviewed and we believe it accurately reflects the descriptive data in the text, and so we have kept the original format.

 We have adjusted Table 2 to avoid duplication and merged the first column cells, and retained Table 1 which we believe to be a useful summary.

 In answer to the question "It is good to use GLMMs, however I did not see results from this analysis. Could you please show and draw out what is the main contribution to the variation of outcome?", the variability estimates for within-day and between-day variation are the outputs of the GLMM, as are all statistical comparisons presented (e.g. p-values).

 Regarding the application of Abbot’s correction to mortality data, as the purpose of the study was to quantify and explain variability in observed outcomes, it was decided to assess the true outcomes rather than an adjusted metric.

# Reviewer report: Reviewer response for version 1

**DOI:** 10.21956/gatesopenres.16785.r37274

**Published:** 2024-08-20

**Authors:** Magellan Tchouakui

**Affiliations:** 1 Centre for Research in Infectious Diseases, Yaoundé, Cameroon

This study aimed to identify variations in bottle bioassay methodologies and assess the potential impact on the data generated because resistance monitoring is a key element in controlling vector-borne diseases.  The authors used a systematic examination of existing literature and protocols from WHO and CDC to evaluate the impact of several variables on bioassay results experimentally. They noticed that two key factors are influencing the outcomes of bioassays including mosquito dry weight and relative humidity (RH). This finding not only advances our comprehension of these assays but also underscores the importance of establishing precisely defined methodologies for resistance monitoring. I fully recommend the acceptance of this article as it will significantly help to strengthen the robustness of bioassays

Is the work clearly and accurately presented and does it cite the current literature?

Yes

If applicable, is the statistical analysis and its interpretation appropriate?

Yes

Are all the source data underlying the results available to ensure full reproducibility?

No source data required

Is the study design appropriate and is the work technically sound?

Yes

Are the conclusions drawn adequately supported by the results?

Yes

Are sufficient details of methods and analysis provided to allow replication by others?

Yes

Reviewer Expertise:

Infectious diseases transmission, Insecticide resistance management, Malaria, Anopheles mosquitoes, Fitness cost

I confirm that I have read this submission and believe that I have an appropriate level of expertise to confirm that it is of an acceptable scientific standard.

# Response

**Published:** 2024-12-11

**Authors:** Rosemary Lees

**Affiliations:** Liverpool School of Tropical Medicine, UK

We are very grateful for this positive response, and are pleased that the reviewer found the article’s findings useful.

# Reviewer report: Reviewer response for version 1

**DOI:** 10.21956/gatesopenres.16785.r37275

**Published:** 2024-07-23

**Authors:** Shüné V Oliver

**Affiliations:** 1 University of the Witwatersrand Johannesburg, Johannesburg, Gauteng, South Africa

The authors present a crucial study that really should have been performed a long time ago. The assessment of insecticide resistance is possibly the most critical aspect of vector control and one that is not to be taken lightly. The authors present a comprehensive literature review and experimental data of what is quite frankly a disturbing array of variability in key methods used for assessing resistance. This paper is a useful and important summary of these problems and will be a useful resource for people in the field. I have a few very small recommendations that would be needed before indexing.

 I will describe my changes:

 Please define the synergist acronym MERO. 

 Page 7: italicize Anopheles, Aedes, Culex. 

 Page 9

 In the results section under the first section Guidelines review: assessing updates and changes in methodology: 

 (Last line of first part of the PDF, going on until the end of section)

 "At the time this new WHO methodology had several drawbacks: the test kit was seen

 to be expensive; test papers were not provided by WHO for certain insecticides; there was no provision for assessment of metabolic resistance; and the recommended diagnostic doses were not appropriate for all species. As a result, CDC looked to develop a bioassay methodology to address these issues29. At

 the time this new WHO methodology had several drawbacks: the test kit was seen to be expensive; test papers were not provided by WHO for certain insecticides; there was no provision for assessment of metabolic resistance; and the recommended diagnostic doses were not appropriate for all species. As a result, CDC looked to develop a bioassay methodology to address these issues".

 Please look at this section carefully again as it seems that a portion of the paragraph was duplicated. If I am wrong, then it is very confusing as I read this section multiple times and it looks like a repeat.

Is the work clearly and accurately presented and does it cite the current literature?

Yes

If applicable, is the statistical analysis and its interpretation appropriate?

Yes

Are all the source data underlying the results available to ensure full reproducibility?

Yes

Is the study design appropriate and is the work technically sound?

Yes

Are the conclusions drawn adequately supported by the results?

Yes

Are sufficient details of methods and analysis provided to allow replication by others?

Yes

Reviewer Expertise:

Entomology, Vector Control, Insecticide resistance, Mosquito biology.

I confirm that I have read this submission and believe that I have an appropriate level of expertise to confirm that it is of an acceptable scientific standard.

# Response

**Published:** 2024-12-16

**Authors:** Rosemary Lees

**Affiliations:** Liverpool School of Tropical Medicine, UK

We are grateful for the positive reception to this article and are pleased that the reviewer also thinks this was a useful exercise. We have addressed all the comments on the article in version 2, and appreciate the reviewer picking up on these points to improve the manuscript.
